# Adult Langerhans Cell Histiocytosis Presenting as an Isolated Rectal Polyp and Hematochezia

**DOI:** 10.7759/cureus.108093

**Published:** 2026-05-01

**Authors:** Liena Zhao, David Driman

**Affiliations:** 1 Department of Pathology and Laboratory Medicine, London Health Sciences Centre, Western University, London, CAN

**Keywords:** colorectal, gastrointestinal, hematochezia, langerhans cell histiocytosis(lch), polyp

## Abstract

Langerhans cell histiocytosis (LCH) is a rare disorder characterized by the abnormal proliferation and accumulation of Langerhans cells, which are specialized dendritic cells involved in antigen presentation. LCH most commonly involves the skin, bone, and lung. The gastrointestinal tract is an uncommon site of disease, and LCH presenting as a solitary colonic polyp is extremely rare. We present a case of a 53-year-old male with hematochezia who had a rectal polyp that was confirmed both histologically and immunohistochemically to be LCH. There was no evidence of systemic involvement of LCH.

## Introduction

Langerhans cell histiocytosis (LCH) is a rare and heterogeneous disorder characterized by the clonal proliferation of myeloid precursor cells that differentiate into pathologic CD1a⁺/CD207⁺ (langerin-positive) Langerhans cells [1,2]. The Langerhans cell is a type of dendritic cell that normally functions in antigen presentation and immune response [2,3]. The incidence of LCH is higher in children than in adults; the average age at diagnosis in adults is around 40 years, and about two-thirds of patients already have involvement of multiple organ systems at the time of diagnosis [3]. Adult LCH may be associated with other neoplastic diseases, especially other myeloproliferative neoplasms [4].

LCH can have variable manifestations, ranging from single-site to multiple sites of disease, most commonly involving bone, skin, and lung [3]. Other sites of LCH have also been reported, including lymph nodes, the nervous system, liver, spleen, and ear [4,5]. The clinical presentation is highly variable, ranging from an acute, fulminant, and disseminated form historically referred to as Letterer-Siwe disease to more localized, indolent, and chronic lesions involving bone or other organs, commonly termed eosinophilic granuloma [3]. Involvement of the gastrointestinal tract is rare in LCH and is typically identified in pediatric patients with systemic disease [1,2]. Solitary gastric or intestinal LCH in adults is extremely rare and may be overlooked or misdiagnosed [6]. This report describes an adult patient with LCH presenting as an isolated rectal polyp with rectal bleeding.

## Case presentation

A 53-year-old male with a medical history of multiple sclerosis, osteoarthritis, rheumatoid arthritis, and psoriasis presented to the hospital for hematochezia. Endoscopy identified a 6 mm descending colon polyp and a 5 mm rectal polyp that were completely removed. The rectal polyp was endoscopically suggestive of a neuroendocrine tumour. No other lesions were noted on colonoscopy. There was no significant abnormality in the esophagus, stomach, or duodenum. No colonoscopy images were obtained. Laboratory tests, including liver enzymes, were normal (Table 1).

**Table 1:** Laboratory test results

Test	Result	Units	Normal range
Alanine aminotransferase (ALT)	20	U/L	<46
Alkaline phosphatase (ALP	64	U/L	40-129
Gamma-glutamyl transferase (GGT)	22	U/L	<65
International normalized ratio (INR)	1.0		0.9-1.2
Ferritin	114	ug/L	15-275
Hemoglobin A1C	5.4	%	<6.0
Creatinine	80	umol/L	60-100
Leukocytes; blood	7.9	x 10^9^/L	4.0-11.0
Erythrocytes; blood	4.51	x 10^12^/L	4.50-6.00
Hemoglobin; blood	155	g/L	135-175
Hematocrit; blood	0.440	L/L	0.400-0.500
Platelets; blood	239	x 10^9^/L	150-400
Neutrophils; blood	6.2	x 10^9^/L	2.0-7.5
Lymphocytes; blood	1.0	x 10^9^/L	1.0-3.5
Monocytes; blood	0.6	x 10^9^/L	0.2-1.0
Eosinophils; blood	0.0	x 10^9^/L	0.0-0.5
Basophils; blood	0.0	x 10^9^/L	0.0-0.2
Granulocytes immature	0.0	x 10^9^/L	0.0-0.1

**Figure 1:**
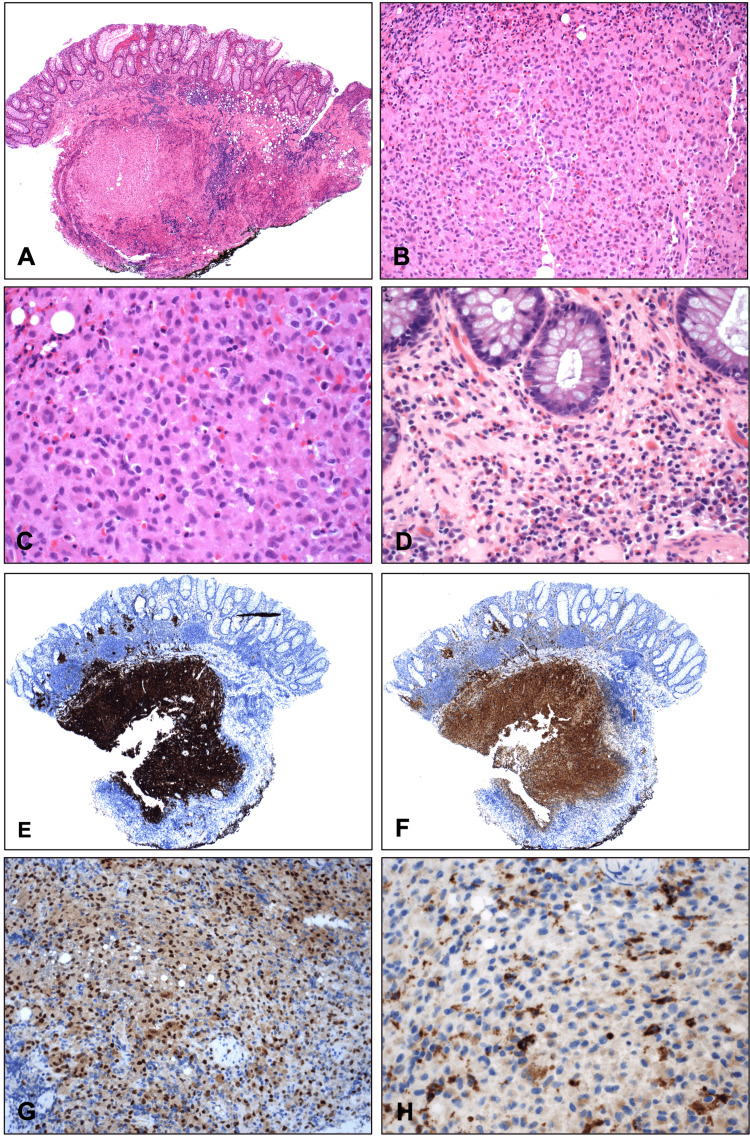
Rectal polyp showing Langerhans cell histiocytosis The rectal polyp shows a polypoid submucosal lesion (A). The lesion is composed of sheets of bland epithelioid cells with many eosinophils and scattered multinucleated giant cells (B). The lesional cells exhibit irregular nuclear contours with nuclear grooves (coffee-bean appearance), fine chromatin, inconspicuous nucleoli, and abundant eosinophilic cytoplasm. There is no mitosis or necrosis. The background consists predominantly of eosinophils with scattered multinucleated histiocytes (C). Abundant eosinophils are also present in the mucosa (D). By immunohistochemistry, the lesional cells are strongly positive for CD1a (E) and S100 (F). There is nuclear immunoreactivity to cyclin D1 (G). There is patchy cytoplasmic positivity for CD68 with Golgi dot-like staining (H). Original magnifications: A: x40; B: x200; C: x400; D: x400; E: x40; F: x40; G: x200; H: x400

Microscopically, the rectal polyp displayed a relatively well-circumscribed submucosal lesion comprised of a mixture of mainly bland polygonal cells with nuclear grooves, coffee bean-shaped nuclei, fine chromatin, inconspicuous nucleoli, and abundant eosinophilic cytoplasm, scattered multinucleated giant cells, as well as abundant eosinophils (Figure 1). The overlying mucosa showed chronic inflammation with prominent eosinophils and reactive changes. There was no epithelial dysplasia. By immunohistochemistry, the lesional cells were diffusely positive for S100, CD1a, and cyclin D1. There was patchy positivity for CD68 with Golgi dot-like staining. CD117, HMB45, pan cytokeratin, synaptophysin, and chromogranin were negative. Along with the morphological features, the immunohistochemical profile confirmed the diagnosis of LCH. The descending polyp was a tubular adenoma. On further workup, there was no evidence of involvement of any other organ system. Molecular testing was not performed on this polyp.

## Discussion

LCH is a rare disorder characterized by the abnormal proliferation and accumulation of Langerhans cells, which are specialized dendritic cells involved in antigen presentation. The disease spectrum is highly variable, ranging from isolated bone lesions to widespread multisystem involvement affecting organs such as the skin, lungs, liver, and hematopoietic system. Although it can occur at any age, it is more commonly diagnosed in children, while adult-onset cases often present differently and may be underrecognized. Gastrointestinal tract involvement by LCH is rare and is typically found in pediatric male patients with systemic disease, and is associated with a poor prognosis [3,7,8]. Isolated gastrointestinal LCH in adult patients presenting as polyps is extremely rare [9,10].

The diagnosis of rectal LCH is often delayed due to its nonspecific presentation and rarity. Patients may present with nonspecific symptoms such as abdominal pain, rectal bleeding, or altered bowel habits, which can easily be mistaken for more common conditions such as hemorrhoids, inflammatory bowel disease, or rectal cancer [6,11,12]. The rarity of rectal involvement in LCH means it is often not included early in the differential diagnosis, leading to potential delays in diagnosis. Our patient had no clinical history of systemic LCH. The patient’s rectal bleeding may have been attributable to the LCH; however, another source is also possible.

In contrast to pediatric cases, LCH in adults more commonly presents as a localized process, frequently involving a single organ system. When the gastrointestinal tract is affected, adult patients typically exhibit unifocal disease confined to sites such as the colon or stomach, often identified incidentally during endoscopic evaluation [13,14]. Adults with an incidental colonic LCH polyp identified on routine colonoscopy are typically asymptomatic, and most lesions encountered on routine endoscopy are described as small, solitary polyps [10,12,15,16]. Few cases of gastric LCH have been described, and none of the reported patients had a documented recurrence or progressed to systemic involvement [17-19]. In one study, two of 10 adult patients progressed to multifocal, multisystem disease involving the skin. However, these cases were considered low risk and responded to treatment [14]. No specific factors have been identified that are associated with disease progression in this setting.

The differential diagnosis of rectal LCH includes other histiocytic lesions such as histiocytic sarcoma, granular cell tumour, melanoma, neuroendocrine tumour, and mastocytosis [20]. The morphological features of gastrointestinal tract LCH are similar to those of LCH at other sites. Immunohistochemistry is essential to confirm the diagnosis, as lesional cells are diffusely positive for CD1a and S100 protein. Other S100 immunoreactive lesions with an epithelioid morphology in the intestine include granular cell tumour, Rosai Dorfman disease, and melanoma. However, their distinctive cytological features should help avoid diagnostic confusion. Melanoma is characterized by prominent nuclear pleomorphism and may contain pigment.

Although S100 can be positive in both LCH and melanoma, melanoma is usually HMB-45 and Melan-A positive and CD1a negative. Mastocytosis can show a range of morphologic appearances resembling histiocytes as well as a marked eosinophilic infiltrate. CD117 negativity and CD1a positivity exclude mastocytosis. Cyclin D1 is a reliable and frequently expressed diagnostic marker in LCH and can be used to help distinguish LCH from reactive Langerhans cells. Infection should also be ruled out; parasitic infection can cause histiocytic aggregates with eosinophils in the colon.

## Conclusions

Although uncommon, isolated LCH may present as a colorectal polyp with rectal bleeding and should be considered among the rare causes of colorectal polyps. Complete excisional biopsy remains crucial to prevent missed diagnosis. Endoscopic concern for a neuroendocrine tumour, combined with histiocytic cells showing nuclear grooves and an eosinophil-rich background, should prompt consideration of LCH. As screening colonoscopy becomes more widely performed, additional cases are likely to be detected, necessitating accurate recognition and diagnosis.
